# Rhophilin-2 Upregulates Glutamine Synthetase by Stabilizing c-Myc Protein and Confers Resistance to Glutamine Deprivation in Lung Cancer

**DOI:** 10.3389/fonc.2020.571384

**Published:** 2021-01-20

**Authors:** Dakai Xiao, Jiaxi He, Zhihua Guo, Huiming He, Shengli Yang, Liyan Huang, Hui Pan, Jianxing He

**Affiliations:** ^1^ Department of Thoracic Surgery, The First Affiliated Hospital of Guangzhou Medical University, Guangzhou, China; ^2^ Guangzhou Institute of Respiratory Disease & State Key Laboratory for Respiratory Disease, Guangzhou, China; ^3^ Research Center for Translational Medicine, The First Affiliated Hospital of Guangzhou Medical University, Guangzhou, China; ^4^ Department of Thoracic Surgery, The First Hospital of Foshan City, Foshan, China

**Keywords:** lung adenocarcinoma, RHPN2, glutamine synthetase, c-Myc protein, tumorigenesis

## Abstract

**Introduction:**

RHPN2, a member of rhophilin family of rho-binding proteins, regulates actin cytoskeleton and vesicular trafficking, and promotes mesenchymal transformation in cancer. We have found that RHPN2 was significantly mutated in lung adenocarcinoma (LUAD). However, the role of RHPN2 in lung cancer is not fully understood.

**Methods:**

In the present study, we investigated the expression of RHPN2 in 125 patients with LUAD by qRT-PCR and correlated its expression with clinical characteristics. The effects of RHPN2 on the proliferation and invasion of lung cancer cells were determined by CCK-8 and *in vitro* transwell assays, clonogenic assay, and xenograft mouse model. The RhoA pull down assay and Western blotting were performed to elucidate the mechanism of RNPN2 in tumorigenesis of lung cancer.

**Results:**

RHPN2 was overexpressed in tumors from LUAD, and high levels of RHPN2 were associated with poor prognosis of LUAD patients. RHPN2 was required for proliferation and invasion of lung cancer cells. Intriguingly, overexpression of RHPN2 conferred the resistance to glutamine depletion in lung cancer cells. Mechanistic studies revealed that ectopic overexpression of RHPN2 promoted the stability of c-Myc protein *via* phosphorylation at Ser62 and increased c-Myc target glutamine synthetase (GS). Analysis of GS expression in clinical sample showed that the expression of GS was elevated in tumor cells. Kaplan-Meier analysis revealed that high levels of GS were significantly associated with worse overall survival time of the patients with LUAD.

**Conclusions:**

Taken together, this study suggested that RHPN2 was involved in tumorigenesis of lung cancer *via* modulating c-Myc stability and the expression of its target GS in lung adenocarcinoma, which links RHPN2 and glutamine metabolism.

## Introduction

Lung cancer remains the leading cause of cancer-related deaths worldwide. With the advance of next generation sequencing technologies, we have gained comprehensive insights into the genetic alterations involved in cancer development and progression. However, the functional role of genetic alterations in cancer is far from clear.

Our previous sequencing analyses of primary lung adenocarcinomas and their corresponding lymph node metastases revealed that several genes involved in cytoskeleton remodeling were significantly mutated or altered in LUAD (1). Among these, RHPN2, a member of Rho-binding family, was significantly mutated and appeared to be a potential driver of LUAD. RHPN2 protein contains several protein interacting domains including an N-terminal HR-1,a centralBro1-like, and a C-terminal PDZ domains (2, 3). The HR-1 domain binds to Rho GTPase, and the Bro1-like domain is required for its recruitment to the late endosome in the presence of activated RhoB (3). The PDZ domain mediates the interaction between RHPN2 and other proteins like KRT18 (3), a component of cytoskeleton. As an effector of Rho GTPase signaling, RHPN2 regulates actin cytoskeletal remodeling, cell migration and invasion (4), and recruitment to late endosome (5). Notably, genome-wide association study (GWAS) revealed that SNP rs10411210 variant in RHPN2 gene was a risk loci for colorectal cancer and was associated with survival outcome (6, 7). Despite these reports, the mechanisms of the action of RHPN2 in cancer are not fully understood.

Glutamine (Gln) is a major source of energy and nitrogen for biosynthesis. Glutaminase (GLS) and Glutamine synthetase (GS, encoded by GLUL gene) are the main enzymes responsible for Gltamine metabolism (8–10). GLS is the rate-limiting enzyme in glutaminolyis and has been shown to be associated with Gln addiction in several types of cancer. GS is responsible for *de novo* glutamine synthesis by catalyzing the condensation of glutamate and ammonia. GS is also necessary for cell adaption to glutamine deprivation. The analysis of GLS and GS expression in clinical samples showed that GLS expression is a poor prognostic factor in TNBC patients (11), and GS was also associated with poor prognosis in ovarian cancer and a subset of hepatocellular carcinoma (12, 13). Furthermore, targeting GS in macrophage and stromal cells inhibits cancer metastasis (14), and GLS inhibition has shown potential anti-cancer activity in preclinical model and clinical studies (15). Additionally, several transcriptional factors such as c-Myc (16), YAP (17), and c-Jun (18) were reported to regulate GLS expression, while c-Myc (19, 20), YAP (21), and beta-catenin (22) were also reported to regulate the transcription of GLUL. In this study, we investigated the expression of RHPN2 in LUAD and its association with clinical features. We found that RHPN2 was required for growth and invasion of lung cancer cells *in vitro.* Interestingly, RHPN2 conferred resistance to glutamine deprivation in lung cancer cells. Further experiments revealed that RHPN2 upregulated the expression of GS protein and its mRNA GLUL *via* modulating the stability of Myc protein. Moreover, IHC analysis showed that the expression of GS was positively associated with worse overall survival time of the patients with LUAD. Collectively, this work demonstrated the tumorigenic role of RHPN2 and identified the GS as a therapeutic target for a subset of lung cancer with a high level of RHPN2.

## Materials and Methods

### Cell Culture and Reagents

Lung cancer cell lines H1299 and A549 were cultured in RPMI-1640 medium supplemented with 10% FBS and Kanamycin/Penicillin. The antibodies against c-Myc, GS, β-actin and Flag were purchased from ABclonal Biotechnology, and RHPN2 antibody was obtained from Abcam.

### Patients and Clinical Information

This study was reviewed and approved by the ethnic committee of the first affiliated Hospital of Guangzhou Medical University. The primary tumors and paired normal tissues were obtained, with written informed consent, from the patients who had undergone surgical resection in our hospital. Once surgically resected, the tissues were snap-frozen and stored in liquid nitrogen. The tumor specimens were evaluated by two independent pathologists to determine the histological subtype and TNM stage. The clinical information was collected, and the status of the patients was followed up approximately every three months over phone.

### Lentivirus-Based shRNA and Expression Vector

To generate a lentiviral expression vector, RHPN2 was amplified from the cDNAs of normal lung tissue with primers containing XbaI and EcoR I cutting sites and tagged with FLAG at C-terminus. The fragment was then ligated into lentiviral expression vector pCDH-CMV-MCS-EF1-GFP-Puro (System Biosciences). The shRNA sequences targeting RHPN2 were synthesized and annealed, and then was inserted into lentiviral-based shRNA vector with HpaI and XhoI restriction cutting sites. The RHPN2 shRNA sequences were as following:

Sense:5’-AACTGGCTTTGTCGAGAGTCGATTCTTCAAGAGAGAATCGACTCTCGACAAAGCCTTTTTTC-3’,

Anti-sense:5’-TCGAGAAAAAAGGCTTTGTCGAGAGTCGATTCTCTCTTGAAGA ATCGACTCTCGACAAAGCCAGTT-3’. The sequence of each construct was confirmed by Sanger sequencing.

### Lentivirus Particle Packaging and Transduction

To generate the lentiviral particles, lentiviral-based shRNA or expression vector, along with packaging vectors psPAX2 and pMD2.G, were transiently transfected into human embryonic kidney cell 293T using the lipofectamine 2000 transfection reagent (Life Technologies, USA). The lentiviruses were transduced into cells in the presence of polybrene (8μg/ml).

### Reverse Transcription PCR (RT-PCR) and Quantitative Real-Time PCR (qPCR)

The total RNAs from lung cancer cells or lung specimens were extracted using Trizol reagent (Life technologies, USA) following the manufacturer’s instruction. The quantity and quality of RNAs were determined by Nanodrop and agarose gel electrophoresis. One microgram of total RNA was used for the synthesis of cDNA using Reverse Transcription System (Promega, USA). Real-time PCR was performed to determine the relative expression of target genes using the Kapa SYBR FAST qPCR kit (KAPA Biosystems, USA). Thermal cycling conditions were as follow: Denaturing for 3 min at 95° followed by 40 cycles at 95° for 3 s and at 60° for 30 s. The quality of the PCR products was monitored using post-PCR melting curve analysis. β-actin was used as an internal control for these measurements. Relative mRNA levels were calculated using 2^-ΔΔCt^ method, in which ΔΔC_t_ represents the mean threshold cycle (Ct) differences between the target gene (C_T_) and that of the β-actin (C_A_) values.

### *In Vitro* Invasion Assay

*In vitro* invasion assay was performed as previously (23). Briefly, 5×10^4^ cells in 200 μl DMEM containing 0.1%FBS and 0.1%BSA were seeded into upper membrane of Transwell inserts (8μm, Corning Costar) coated with Matrigel (BD Biosciences). After 24 h, the cells invaded onto the lower membrane were stained with Wright and Giemsa Stain and cell number was counted with ImageJ software.

### *In Vitro* Clonogenic Assay

Clonogenic assay was performed as previously (23). Briefly, 200 cells were plated into 6-well plates. After 10 days, cells were washed with PBS two times and fixed with methanol/acetic acid (3:1) and stained with 0.5% crystal violet.

### Immunoblotting

Equal amounts of protein extracts from all samples were applied to SDS-PAGE and then transferred to PVDF membrane (Millipore). The membrane was blocked in 5% nonfat milk in Tris Buffered Saline buffer with 0.1%Tween-20 (TBST) followed by incubation with the primary antibody at 4° overnight. After incubation with corresponding secondary antibodies for 2hrs at room temperature, bands were visualized using the enhanced chemiluminescence system (Bio-Rad) and images were captured by X-film or using a CCD camera (Tanon, China).

### RhoA Pulldown Activation Assay

RhoA activation assay was performed according to the manufacturer’s instructions (Cytoskeleton, USA). In brief, after an initial growth in complete medium cells were cultured overnight in a serum-free medium. Then the cells were stimulated with 10%FBS for 10 min. Following this, cells were harvested and lysed in cold lysis buffer. Equivalent protein amounts of lysate were used for the pulldown assays. The samples were then subjected to SDS-PAGE and Immunoblotting to detect the RhoA activity.

### Tumor Growth Experiments

Four- to 6-week-old BALB/c male nude mice were used to assess the *in vivo* tumorigenic ability of human lung cancer A549 cells expressing RHPN2 and empty vector. Cells were suspended in serum-free RPMI-1640 and then subcutaneously injected into the right inguinal fold of mice (2×10^6^cells/mouse). Tumor size was measured with calipers three times a week, and tumor volume was estimated by the formula: V=1/2 × length × width^2^. Mice were euthanized and sacrificed when the tumor size reached ~1000mm^3^. Tumors were removed and weighed.

### Statistical Analyses

The differences of RHPN2 mRNA levels between tumors and corresponding normal tissues were determined by paired student’s *t* test. The correlations of RHPN2 expression with clinical features were determined by the Pearson’s χ^2^ test. The overall survival of the patients was analyzed by the Kaplan–Meier curve with the log-rank test. P < 0.05 was considered to be significant. All statistical analyses were performed using Prism 5 (GraphPad) and SPSS16.0.

## Results

### RHPN2 Was Dysregulated in the Patients With Lung Adenocarcinoma and Higher Levels of RHPN2 Were Associated With Worse Overall Survival Time

**Figure 1:**
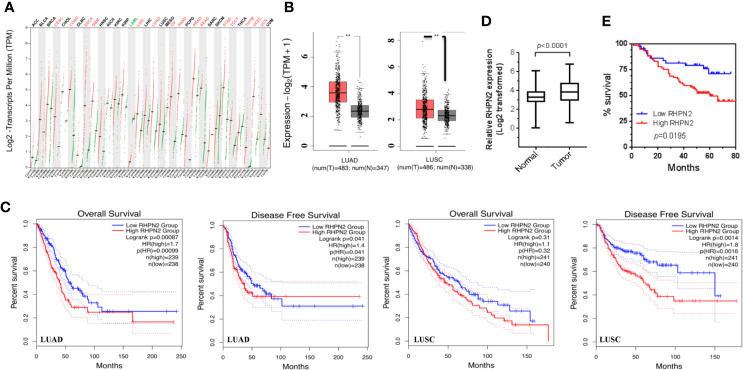
Overexpression of RHPN2 is associated with worse clinical outcome of patients with lung adenocarcinoma. **(A)** The log_2_-transformed expression of RHPN2 in primary tumors and normal tissues of the patients with lung cancer and other cancer types from TCGA. **(B)** The log2 transformed expression of RHPN2 in tumors and normal tissues of lung adenocarcinoma (LUAD) and LUSC from TCGA. **(C)** The association of RHPN2 levels with overall survival and disease-free survival in LUAD and LUSC from TCGA. **(D)** The relative expression of RHPN2 in tumors and matched normal tissues from our institute. **(E)** Overall survival curves for the LUAD patients from our institute with different levels of RHPN2. ** p < 0.01 compared with control group.

We have performed RNA sequencing of the tumor tissues and matched normal counterparts from LUAD patients previously (1). We reanalyzed the RNA-seq data and found that RHPN2 was highly expressed in LUAD tumors (*p*<0.0001, **Figure S1**), with 44/59 (74.6%) patients having higher levels of RHPN2 mRNA in tumor tissues than the normal counterparts. To validate the RHPN2 expression in LUAD and other cancer types, we obtained the RNA sequencing data and corresponding clinical information from TCGA and found that RHPN2 mRNA levels were higher in tumor tissues than normal tissues in LUAD, LUSC and other cancer types (**Figures 1A, B**). Moreover, we also found that the higher levels of RHPN2 was associated with shorter disease-free survival in LUAD (Logrank test, p=0.041) and LUSC (*p*=0.0014) and shorter overall survival (p=0.00087) in LUAD (**Figure 1C**). To further validate these results in LUAD, we continued to examine the RHPN2 mRNA levels in an independent cohort of 125 LUAD patients from our institute with follow up information available. Consistently, RHPN2 mRNA was highly expressed in tumors (*p*<0.0001, **Figure 1D**), with 78/125 (62.4%) patients having higher levels of RHPN2 mRNA in tumors than normal tissues. Importantly, Kaplan-Meier survival analysis revealed that higher levels of RHPN2 were associated with poor clinical outcome (log rank test, *p*=0.0195, **Figure 1E**). However, Pearson’s Chi-square analysis showed that there were no significant associations between RHPN2 expression levels and clinical features of the LUAD patients such as age, gender, smoking status, TNM stage and lymph node metastasis (**Table S1**). These results suggested that RHPN2 was frequently altered in LUAD patients and higher levels of RHPN2 were associated with worse clinical outcomes.

### RHPN2 Is Required for Cell Growth and Invasion of Lung Cancer Cells

**Figure 2:**
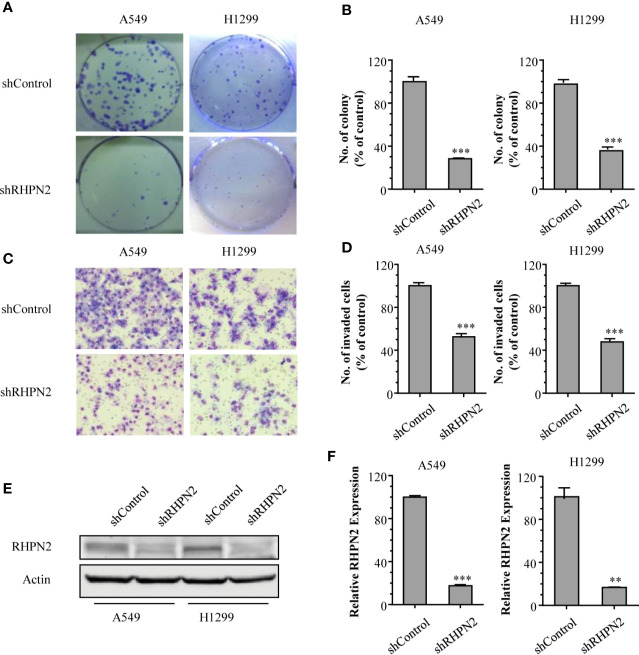
RHPN2 is required for cell growth and *in vitro* invasion of lung cancer cells. **(A, B)** the clonogenic formation assay was performed to detect the growth of A549 and H1299 cells stably expressing shControl and shRHPN2. The representative images and quantitative results were shown in **(A, B)**, respectively. **(C, D)** Transwell invasion assay was used to examine invasive ability of A549 and H1299 cells. The representative images and quantitative results of invaded cells in A549 and H1299 cells were shown in **(C, D)**, respectively. **(E, F)** the reduced expression of RHPN2 in RHPN2-depleted A549 and H1299 cells was confirmed by immunoblotting **(E)** and RT-qPCR **(F)**. The data were representative of three independent experiments and were expressed as mean ± SD. **p < 0.01 and ***p < 0.0001 compared with control group.

To explore the biological function of RHPN2 in lung cancer, we generated lung cancer cells expressing low level of RHPN2 through lentivirus-mediated short hairpin RNA (shRNA). The reduction of RHPN2 protein and mRNA was confirmed by RT-qPCR and immunoblotting, respectively (**Figures 2E, F**). We further examined the capacity of growth and *in vitro* invasion of lung cancer cells expressing shRNPN2 and shControl with clonogenic formation assay and transwell assay, respectively. As shown in **Figures 2A–D**, RHPN2 depletion significantly impaired the clonogenic and invasive ability of A549 and H1299 cells expressing shRHPN2 and shControl. These data indicated that RHPN2 was required for the growth and invasion of lung cancer cells.

### RHPN2 Promotes Tumorigenesis of Lung Cancer *In Vivo*

**Figure 3:**
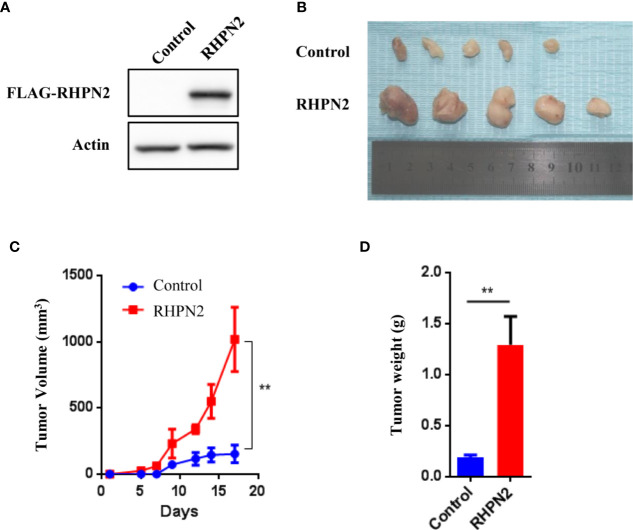
RHPN2 promotes growth of lung cancer cells *in vivo*. **(A)** The expression of FLAG-tagged RHPN2 in A549 cells was determined by Western Blotting using anti-FLAG antibody. **(B)** The tumors formed in nude mice injected by A549 cells expressing FLAG-RHPN2 and empty vector. **(C, D)** Quantitative results of tumor weight **(C)** and tumor volume **(D)**. Tumor volume was measured three times every week. The data were shown as mean ± SD. ** p < 0.01 compared with vector control.

Since RHPN2 was required for the growth and invasion of lung cancer cells, we continued to determine the effect of RHPN2 on the growth of lung cancer *in vitro* and *in vivo*. We generated lung cancer cells expressing the FLAG-tagged RHPN2 (**Figure 3A**). However, RHPN2 overexpression has no effect on the growth of A549 cells determined by colony formation assay (data not shown). In contrast, A549 cells stably transduced with FLAG-tagged RHPN2 were subcutaneously inoculated into nude mice, tumors started to appear within 1 week in mice. A dramatic increase in tumor volume and weight was observed in RHPN2 compared to the vector control (**Figures 3B–D**, *p*<0.05). Taken together, these experiments suggested that RHPN2 promoted tumorigenesis *in vivo*.

### RHPN2 Suppressed RhoA Activation

**Figure 4:**
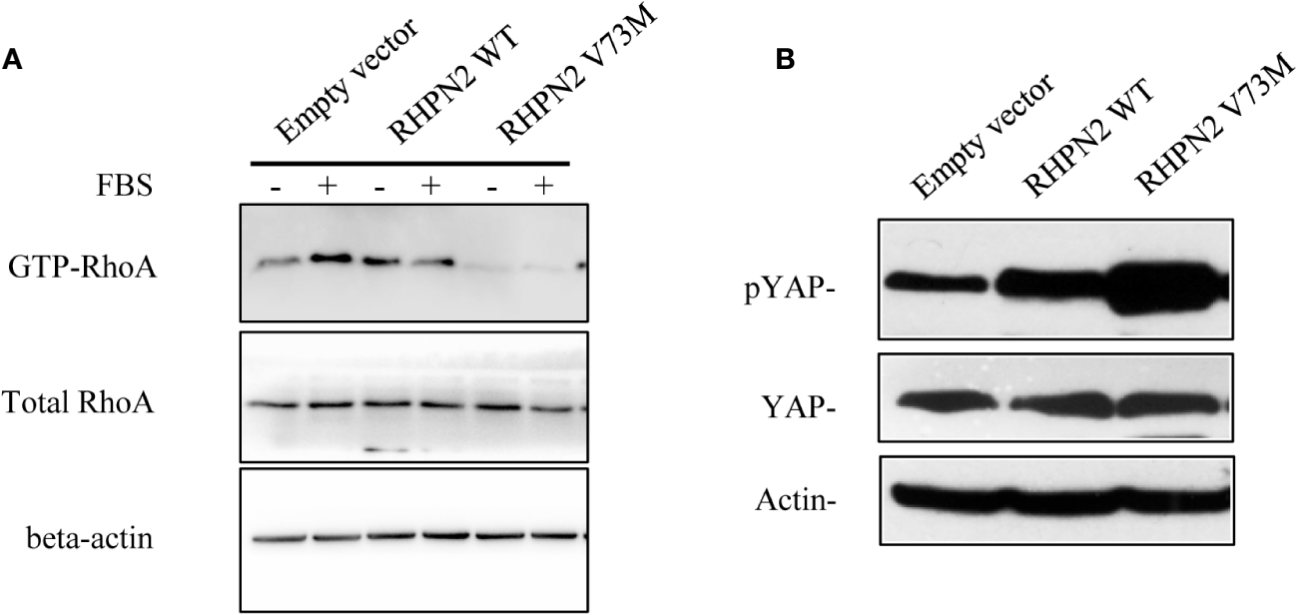
RHPN2 suppresses RhoA signaling. **(A)** Western blotting showing the expression levels of active RhoA (Rho-GTP) and total RhoA in A549 cells expressing empty vector, WT and mutant RHPN2 with or without serum stimulation. Expression of β-actin serves as loading control. **(B)** Western blot showing the expression of YAP and p-YAP in A549 cells expressing empty vector, WT and mutant RHPN2. Expression of β-actin serves as loading control.

It was reported that RHPN2 bound RhoA and regulated its GTPase activity (4), and active RhoA could induce dephosphorylation of YAP, which was a key effector of Hippo pathway (24). Thus, we tested whether RHPN2 could affect RhoA activity and subsequently regulate Hippo-YAP pathway in lung cancer cells. To investigate the effect of RHPN2 on the RhoA activity, we performed RhoA pull-down activation assay in RHPN2-expressing lung cancer cells. As expected, serum stimulated the RhoA activity in A549 cells expressing empty vector (**Figure 4A**, lane 1 vs lane 2). However, we did not observe the stimulation of RhoA activity in RHPN2-expressing A549 cells even in the presence of serum (**Figure 4A** lane 3 vs lane 4). In contrast, point mutant of RHPN2 within Rho-binding domain(V73M) not only abolished the basal RhoA activity in the absence of serum, but also blocked the serum-induced RhoA activation. As constitutively active RhoA-L63 mutant induced YAP dephosphorylation and conversely, Rho GTPases inhibitor elevated phosphorylation of YAP/TAZ (25), we continued to examine the effect of RHPN2 on the YAP activity (**Figure 4B**). Notably, consistent with its inhibitory effect on serum-induced RhoA activity, RHPN2 significantly increased the level of of YAP phosphorylation at serine 127. In contrast to previous study (4), these data suggested that RHPN2 attenuated RhoA activity triggered by serum and subsequently activated Hippo signaling evidenced by higher level of p-YAP.

### RHPN2 Regulates Glutamine Synthetase and Confer Resistance to Glutamine Depletion in Lung Cancer Cells

**Figure 5:**
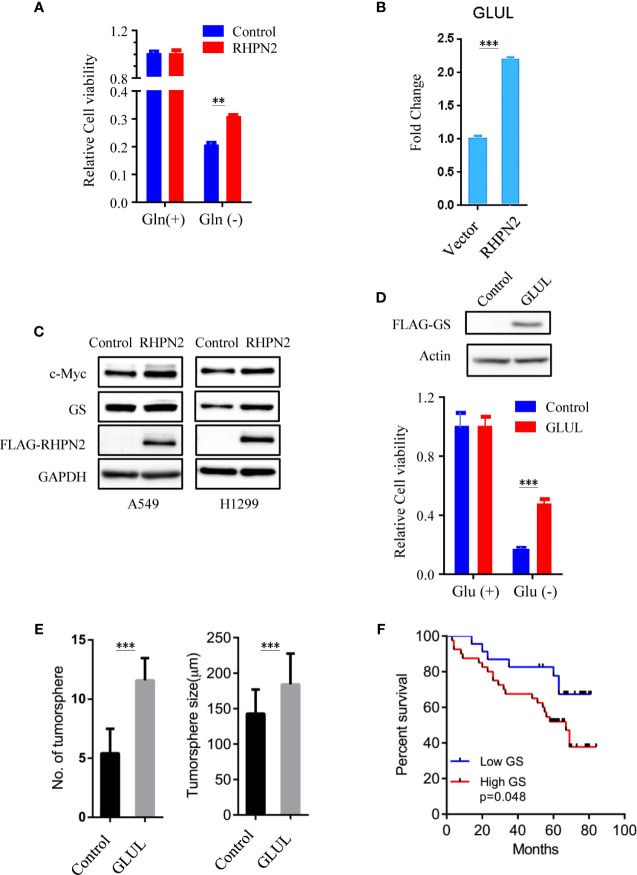
RHPN2 upregulates GLUL expression and confers sensitivity to glutamine deprivation. **(A)** MTT assay to assess the proliferation of A549 cells stably expressing FLAG-RHPN2 or Vector control in the presence or absence of glutamine. **(B)** RT-qPCR analysis of expression of GLUL in A549 cells stably expressing RHPN2 or vector control. **(C)** Immunobloting analysis of cell lysates from A549 and H1299 cells stably expressing RHPN2 or vector control. **(D)** MTT assay to assess the proliferation of A549 cells stably expressing FLAG-GLUL or Vector control in the presence or absence of glutamine. **(E)** Quantitative results of tumorsphere number and size of H1299 cells expressing GLUL or vector control. **(F)** Kaplan-Meier overall survival curves for the patients with lung adenocarcinoma with different levels of glutamine synthetase (GS). **p < 0.01 and ***p < 0.0001 compared with empty vector.

**Figure 6:**
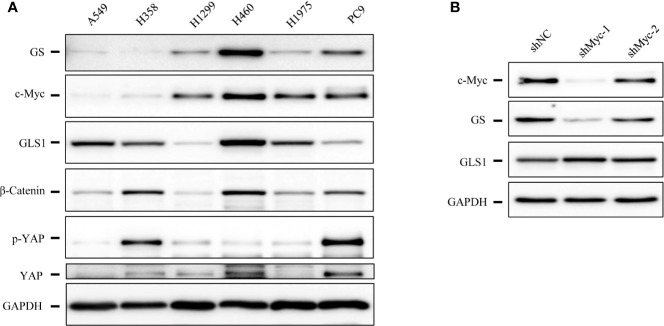
The expression levels of glutamine synthetase (GS) were correlated with that of c-Myc in lung cancer cells. **(A)** Immunoblotting analysis of cell lysates of lung cancer cells. **(B)** Immunoblotting analysis of cell lysates of A549 cells expressing shcMyc and shControl.

As RHPN2 enhanced the growth of lung cancer *in vivo*, but not *in vitro*, we suspected that the growth environment specifically enhanced the proliferation of RHPN2-expressing lung cancer cells *in vitro*. To test this, we grew the RHPN2 and Vector-expressing lung cancer cells under glutamine deprivation conditions. Interestingly, MTT assay revealed that RHPN2-expressing A549 cells showed more resistance to glutamine depletion (**Figure 5A**). To test if RHPN2 had any impact on glutamine metabolism, we performed qRT-PCR to determine the expression of enzymes responsible for glutamine metabolism. As shown in **Figures 5B, C**, qRT-PCR analysis and immunoblotting assay revealed that RHPN2 increased expression of GLUL (encoding GS), but not GLS1 (data not shown). Previous study demonstrated that GS fueled *de novo* purine biosynthesis to support the growth of Gln-restricted Glioblastoma (26). Consistently, GS-overexpressing A549 cells were resistant to Gln deprivation (**Figure 5D**). Due to low capacity of tumorsphere formation of A549 cells, we chose H1299 cells to determine the effect of GLUL on the capacity of tumorsphere formation. As shown in **Figure 5E**, GS enhanced the capacity of tumorsphere formation in H1299 cells. We further investigated the clinical significance of GS in tumor tissues from the patients with lung adenocarcinoma. IHC analysis showed GS staining was predominately observed in tumor cells. Kaplan-Meier analysis showed that the higher levels of GS expression were associated with poor clinical outcomes of the patients with lung adenocarcinoma (**Figure 5F**). The expression of GLUL was modulated by several oncogenes, such as c-Myc (19, 20), YAP (21), FOXO (27) and beta-catenin (22). To determine the potential upstream transcriptional factor mediated the RHPN2-induced upregulation of GLUL, we firstly examined the expression of GS and its potential transcriptional factors c-Myc, beta-catenin and YAP in a panel of lung cancer cell lines (**Figure 6A**). The immunoblotting analysis revealed that GS expression was more likely to correlate with c-Myc expression in lung cancer cells. To confirm the correlation between the expression of GS and c-Myc, we knocked down c-Myc expression in A549 cells using lentivirus-based shRNA. The expression of GS was dramatically reduced in A549 cells transduced with high knockdown efficiency of c-Myc shRNA, but not in those with low knockdown efficiency (**Figure 6B**). This result suggested that GS was transcriptionally regulated by c-Myc. Of note, the reduced expression of c-Myc had no impact on the expression of GLS1(**Figure 6B**), which was reported as a potent target of c-Myc previously (28).

### RHPN2 Modulates the Stability of c-Myc Protein

**Figure 7:**
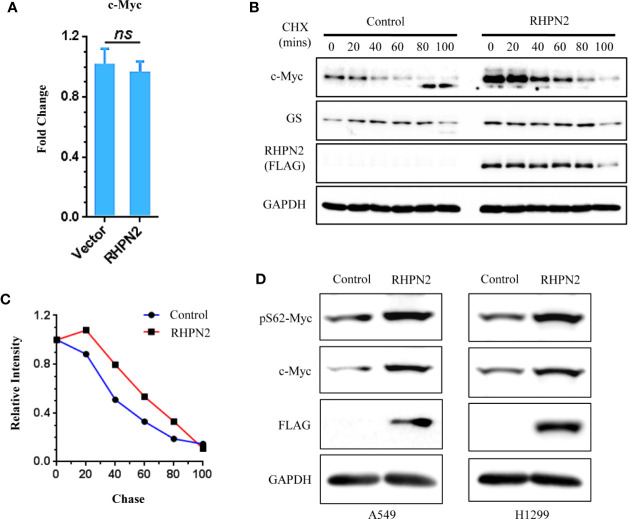
RHPN2 stabilizes c-Myc protein. **(A)** RT-qPCR analysis of expression of MYC in A549 cells expressing RHPN2. **(B)** CHX chase assay analysis of c-Myc degradation in A549 cells expressing RHPN2 or vector control. **(C)** Quantification of the levels of c-Myc normalized by the intensity of GAPDH in **(B)** using ImageJ. **(D)** Immunoblotting analysis of the expression of c-Myc and pSer62-Myc in A549 and H1299 cells expressing RHPN2 or vector control. ns, not significant.

We further examined the impact of RHPN2 on the expression of c-Myc. RHPN2 upregulated c-Myc protein (**Figure 5C**), however, RHPN2 had no impact on the expression of c-Myc mRNA (**Figure 7A**), which led us to examine the effect of RHPN2 on the c-Myc degradation. CHX chase assay showed that RHPN2 remarkably retarded the degradation of c-Myc protein, which suggested that RHPN2 promoted the stability of c-Myc in lung cancer (**Figures 7B, C**). As the stabilization of c-Myc protein is enhanced by the phosphorylation of c-Myc at Ser62 (29), we continued to examine the levels of phosp-Myc in lung cancer cells. Immunoblotting assay revealed that RHPN2 overexpression upregulated the levels of phosp-Myc at Ser62 (**Figure 7D**), which suggested that RHPN2 might regulate the stabilization of c-Myc *via* phosphorylation of c-Myc at Ser62 in lung cancer cells.

## Discussion

RHPN2 has been shown to regulate actin cytoskeleton and promotes mesenchymal transformation. However, the role of RHPN2 in lung cancer remains unclear. Our previous work has demonstrated that RHPN2 was significantly either mutated or overexpressed in human lung adenocarcinomas. In this study, we have provided evidence that RHPN2 promotes lung tumorigenesis. Specifically, we found that higher levels of RHPN2 were associated with poor clinical outcome of patients with lung adenocarcinoma. Moreover, RHPN2 was also required for the growth and invasion of lung cancer cells *in vitro* and promoted tumorigenesis of lung cancer *in vivo*. Furthermore, lung cancer cells expressing high level of RHPN2 was resistance to glutamine depletion. Mechanistically, we demonstrated that RHPN2 upregulated the expression of glutamine synthetase through stabilizing the transcriptional factor c-Myc.

RHOA is a member of RHO family small GTPases. A large body of evidence has demonstrated that activation of RHOA promotes tumorigenesis (30, 31). RHPN2 was identified as a binding partner of RhoA, and enhanced RhoA activity previously (2, 4). In contrast to these reports, we found the RHPN2 could not activate RhoA, instead, RHPN2 attenuated RhoA activation by serum stimulation. Recently several groups reported frequent somatic RhoA mutations in angioimmunoblastic T cell lymphoma (AITL) and diffuse-type gastric carcinoma (32–36). Functional studies demonstrated these RhoA mutants decreased RhoA activity, which suggests that defective RhoA function might play a role in tumorigenesis. Further functional and biological studies demonstrate that RhoA mutation prevented anoikis in organoid model and expression of Rhoa G17V in CD4+ T cells drives proliferation and T follicular helper (Tfh) cells polarization in transgenic animal model (35, 37). Based on these studies, we speculate that RHPN2 overexpression or mutation might promote tumorigenesis directly *via* modulating RhoA signaling. Furthermore, RhoA was reported to mediate G-protein-coupled receptor signaling *via* regulating the phosphorylation of YAP (25). RhoA also function as upstream of the striatin (STRN)-interacting phosphatase and kinase (STRIPAK) complex to control Hippo signaling and dephosphorylate YAP. It has been shown that RHPN1 requires Kibra/NF2 to induce YAP phosphorylation. However, it was found that RHPN2 could not interact Kibra. Consistent with these findings, RHPN2 also increased YAP phosphorylation at Ser127. It is well known that YAP functions as an oncoprotein and phosphorylation and Inactivation of YAP is assumed to exert tumor suppressing effect. However, we also noticed that YAP acts as tumor suppressor in several cancer types including lung squamous cell carcinoma, breast cancer and haematological cancers, which imply that YAP may exert dual roles in tumor progression depending on cellular context. In our study, we observed that RHPN2 affects both Hippo-YAP signaling and Myc-GLS pathway. However, the exact role of YAP in RHPN2-promoting tumorigenesis remains to be studied further. Another report also challenged that RHPN2 could not interact with any form of RhoA (3). Thus, the effects of RHPN2 on RhoA activity and YAP phosphorylation and their mechanisms require further investigation.

c-Myc is an important transcriptional factor and regulates a diversity of fundamental cellular processes. Through CHX chase assay, we have found that RHPN2 stabilized c-Myc protein *via* phosphorylation of c-Myc at Ser62 in lung cancer. However, c-Myc and RHPN2 are localized in different cell components, c-Myc is mainly detected in nucleus, whereas RHPN2 is localized in cytoplasm and endosome. Thus, we speculated that RHPN2 regulated the stability of c-Myc protein through indirect manner. Indeed, previous studies have identified several kinases includingERK1, JNK and CDKs to phosphorylate c-Myc at Ser62 to enhance the stability of c-Myc (38–40). Therefore, the detailed mechanisms underlying the stabilization of c-Myc by RHPN2 remains further investigation.

Taken together, in this study, we demonstrated that rhophilin protein RHPN2 regulates GS expression *via* stabilizing c-Myc protein and promotes tumorigenesis. Our findings also suggest GS is a potential therapeutic target for those patients with high level of RHPN2 in the tumors.

## Data Availability Statement

The raw data supporting the conclusions of this article will be made available by the authors, without undue reservation.

## Ethics Statement

This study was reviewed and approved by the ethic committee of the first affiliated Hospital of Guangzhou Medical University. The primary tumors and paired normal tissues were obtained, with written informed consent, from the patients who had undergone surgical resection in our hospital.

## Author Contributions

DX, Jianx He, and ZG conceived and designed this project and wrote the manuscript. DX, Jiax He, HH, LH, and SY performed the experiments and analyzed the data. PH collected and analyzed the clinical data. All authors contributed to the article and approved the submitted version.

## Funding

This work was supported by the National Natural Science Foundation of China (DX: No.81101681 and ZG: No.81902312) and Natural Science Foundation of Guangdong Province (DX: No. 2016A030313721 and 2017A030313484). The funders had no role in study design, collection, analysis, and interpretation of data, and in the writing the manuscript.

## Conflict of Interest

The authors declare that the research was conducted in the absence of any commercial or financial relationships that could be construed as a potential conflict of interest.

## Supplementary Material

Supplementary Figure S1 The Log2-transformed expression of RHPN2 in primary tumor compared to those in the matched normal tissues from the patients with lung adenocarcinoma.
