# Supplementary figures and images for: Rhophilin-2 Upregulates Glutamine Synthetase by Stabilizing c-Myc Protein and Confers Resistance to Glutamine Deprivation in Lung Cancer
Source: Front Oncol. 2021 Jan 20;10:571384. doi: 10.3389/fonc.2020.571384 (PMC7855701; doi:10.3389/fonc.2020.571384)

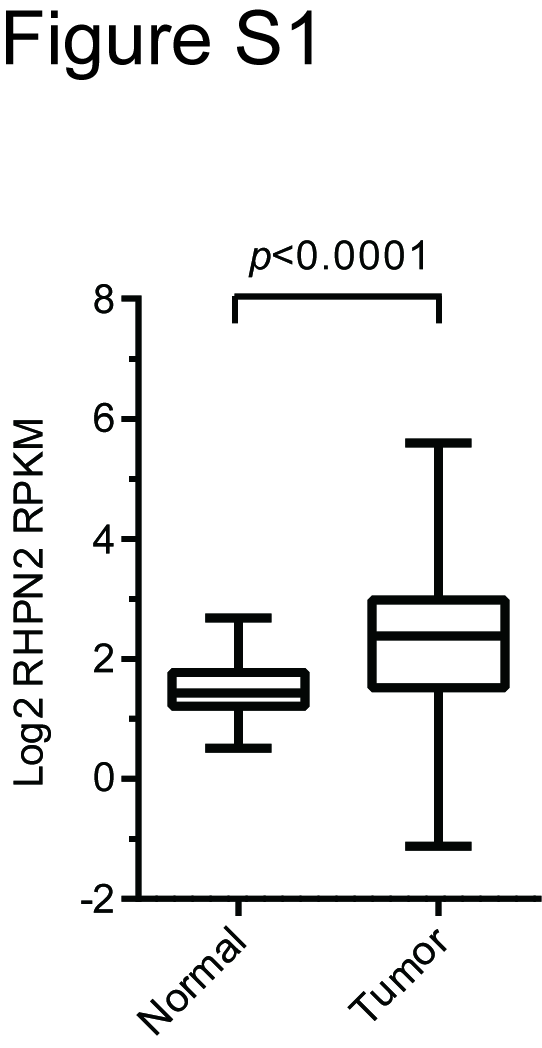

Supplement: Supplementary Figure S1 — The Log2-transformed expression of RHPN2 in primary tumor compared to those in the matched normal tissues from the patients with lung adenocarcinoma. [file Image_1.tif]
