# Supplementary material for: Rhophilin-2 Upregulates Glutamine Synthetase by Stabilizing c-Myc Protein and Confers Resistance to Glutamine Deprivation in Lung Cancer
Source: Front Oncol. 2021 Jan 20;10:571384. doi: 10.3389/fonc.2020.571384 (PMC7855701; doi:10.3389/fonc.2020.571384)
Supplement: Supplementary file 2 [file Table_1.docx]

Table S1. The association of RHPN2 mRNA expression level with the clinical characteristics of patients with lung adenocarcinoma (n=125)

|  |  | RHPN2 Expression | | *p* value |
| --- | --- | --- | --- | --- |
|  |  | Low | High |  |
| Age (year) | Median  range | 57.5  38.7-84.9 | 59.2  38.8-83.4 | 0.396 |
| Gender, n (%) | Male | 24 (19.2) | 40 (32) | 0.981 |
|  | Female | 23 (18.4) | 38 (30.4) |  |
| Smoking status, n (%) | Yes | 13 (10.4) | 23 (18.4) | 0.571 |
|  | No | 32 (25.6) | 54 (43.2) |  |
|  | NA | 2 (1.6) | 1 (0.8) |  |
| TNM stage, n (%) | Ⅰ/Ⅱ | 22 (17.6) | 47 (37.6) | 0.176 |
|  | Ⅲ/Ⅳ | 24 (19.2) | 31 (24.8) |  |
|  | NA | 1 (0.8) | 0 |  |
| Lymph node metastasis, n (%) | Yes | 29 (23.2) | 46 (36.8) | 0.938 |
|  | No | 16 (12.8) | 29 (23.2) |  |
|  | NA | 2 (1.6) | 3 (2.4) |  |
